# Firearm Homicides and Suicides in Major Metropolitan Areas — United States, 2015–2016 and 2018–2019

**DOI:** 10.15585/mmwr.mm7101a3

**Published:** 2022-01-07

**Authors:** Scott R. Kegler, Deborah M. Stone, James A. Mercy, Linda L. Dahlberg

**Affiliations:** ^1^Division of Injury Prevention, National Center for Injury Prevention and Control, CDC; ^2^Division of Violence Prevention, National Center for Injury Prevention and Control, CDC.

Firearm homicides and suicides represent an ongoing public health concern in the United States. During 2018–2019, a total of 28,372 firearm homicides (including 3,612 [13%] among youths and young adults aged 10–19 years [youths]) and 48,372 firearm suicides (including 2,463 [5%] among youths) occurred among U.S. residents (*1*). This report is the fourth in a series* that provides statistics on firearm homicides and suicides in major metropolitan areas. As with earlier reports, this report provides a special focus on youth violence, including suicide, recognizing the magnitude of the problem and the importance of early prevention efforts. Firearm homicide and suicide rates were calculated for the 50 most populous U.S. metropolitan statistical areas (MSAs)^†^ for the periods 2015–2016 and 2018–2019, separated by a transition year (2017), using mortality data from the National Vital Statistics System (NVSS) and population data from the U.S. Census Bureau. Following a period of decreased firearm homicide rates among persons of all ages after 2006–2007 in large metropolitan areas collectively and nationally, by 2015–2016 rates had returned to levels comparable to those observed a decade earlier and remained nearly unchanged as of 2018–2019. Firearm suicide rates among persons aged ≥10 years have continued to increase in large MSAs collectively as well as nationally. Although the youth firearm suicide rate remained much lower than the overall rate, the youth rate nationally also continued to increase, most notably outside of large MSAs. The findings in this report underscore a continued and urgent need for a comprehensive approach to prevention. This includes efforts to prevent firearm homicide and suicide in the first place and support individual persons and communities at increased risk, as well as lessening harms after firearm homicide and suicide have occurred.

NVSS mortality data for 2015–2016 and 2018–2019 were used to identify firearm homicides (*International Classification of Diseases, Tenth Revision* underlying cause codes X93–X95 and U01.4) and firearm suicides (codes X72–X74) among U.S. residents. Firearm homicide and suicide counts were tabulated for the 50 largest MSAs (by population rank midyear 2019).^§^ Tabulated counts were integrated with U.S. Census Bureau population estimates for these MSAs to calculate annual firearm homicide rates for persons of all ages and annual firearm suicide rates for persons aged ≥10 years (data for persons aged <10 years were excluded because suicide intent is often not attributed to young children). Rates were similarly calculated for youths and young adults aged 10–19 years. Overall rates were age-adjusted to the year 2000 U.S. standard population profile. MSA-specific data involving firearm homicide or suicide counts less than 20 are not presented because of concerns related to statistical stability and data privacy. However, such data were included in calculations for the large MSAs combined. This activity was reviewed by CDC and was conducted consistent with applicable federal law and CDC policy.^¶^

Firearm homicide rates among persons of all ages during 2018–2019 varied widely across large MSAs, ranging from 1.1 (the Providence, Warwick [Rhode Island, Massachusetts] MSA and the San Jose, Sunnyvale, Santa Clara [California] MSA) to 18.9 (the Memphis [Tennessee, Mississippi, Arkansas] MSA) per 100,000 residents per year (Table). The rate for all large MSAs combined was 4.8 and the national rate was 4.5, comparable to rates of 4.9 and 4.4 observed for 2015–2016, respectively. The youth firearm homicide rate for the large MSAs combined was 4.9 during 2018–2019 and the national rate was 4.3, both representing increases from 2015–2016 when these rates were 4.7 and 3.9, respectively. Males accounted for approximately 85% of firearm homicide victims during both reporting periods for the 50 largest MSAs combined as well as nationally (National Vital Statistics System, unpublished data, 2020). During both periods, non-Hispanic Black (Black) persons represented a disproportionately large percentage of firearm homicide victims for the large MSAs combined (approximately 65%) relative to population representation (approximately 15%), with a comparable pattern seen nationally (58% and 13%, respectively) (National Vital Statistics System, unpublished data, 2020).

**TABLE:** Numbers and annual rates of firearm homicides and suicides (per 100,000 persons) for the 50 most populous metropolitan statistical areas — United States, 2015–2016 and 2018–2019*

MSA	Yrs	No.^†^ (rate)^§^			
		Firearm homicides		Firearm suicides	
		All ages	Ages 10–19 yrs	Ages ≥10 yrs	Ages 10–19 yrs
**Total**	**2015–2016**	**27,392 (4.4)**	**3,224 (3.9)**	**44,950 (7.7)**	**2,118 (2.5)**
	**2018–2019**	**28,370 (4.5)**	**3,612 (4.3)**	**48,371 (8.1)**	**2,463 (2.9)**
**50 most populous MSAs combined**	**2015–2016**	**17,097 (4.9)**	**2,147 (4.7)**	**18,433 (5.8)**	**850 (1.9)**
	**2018–2019**	**17,027 (4.8)**	**2,259 (4.9)**	**20,122 (6.2)**	**931 (2.0)**
Atlanta, Sandy Springs, Alpharetta (Georgia)	2015–2016	717 (6.3)	106 (6.5)	764 (7.6)	48 (2.9)
	2018–2019	763 (6.5)	108 (6.4)	963 (9.2)	51 (3.0)
Austin, Round Rock, Georgetown (Texas)	2015–2016	99 (2.3)	—^¶^	283 (8.2)	—
	2018–2019	84 (1.9)	—	311 (8.1)	—
Baltimore, Columbia, Towson (Maryland)	2015–2016	656 (12.2)	63 (9.1)	239 (4.7)	—
	2018–2019	676 (12.5)	67 (9.7)	248 (4.7)	—
Birmingham, Hoover (Alabama)	2015–2016	266 (12.8)	23 (8.3)	222 (11.4)	—
	2018–2019	312 (15.1)	32 (11.5)	216 (11.3)	—
Boston, Cambridge, Newton (Massachusetts, New Hampshire)	2015–2016	113 (1.2)	—	179 (2.0)	—
	2018–2019	130 (1.3)	—	189 (2.0)	—
Buffalo, Cheektowaga (New York)	2015–2016	81 (3.6)	—	76 (3.3)	—
	2018–2019	91 (4.2)	—	73 (3.4)	—
Charlotte, Concord, Gastonia (North Carolina, South Carolina)	2015–2016	238 (4.9)	24 (3.6)	352 (8.1)	23 (3.4)
	2018–2019	253 (5.0)	31 (4.4)	367 (7.8)	—
Chicago, Naperville, Elgin (Illinois, Indiana, Wisconsin)	2015–2016	1,527 (8.1)	272 (10.7)	620 (3.6)	29 (1.1)
	2018–2019	1,413 (7.6)	242 (9.8)	666 (3.9)	27 (1.1)
Cincinnati (Ohio, Kentucky, Indiana)	2015–2016	175 (4.1)	31 (5.2)	313 (8.2)	22 (3.7)
	2018–2019	192 (4.5)	34 (5.7)	357 (9.1)	—
Cleveland, Elyria (Ohio)	2015–2016	298 (7.8)	33 (6.4)	277 (7.2)	—
	2018–2019	284 (7.5)	42 (8.4)	315 (8.2)	—
Columbus (Ohio)	2015–2016	206 (5.0)	33 (6.2)	256 (7.0)	—
	2018–2019	205 (4.8)	23 (4.2)	276 (7.3)	—
Dallas, Fort Worth, Arlington (Texas)	2015–2016	537 (3.8)	62 (3.0)	932 (7.8)	54 (2.6)
	2018–2019	613 (4.0)	95 (4.4)	1,074 (8.4)	66 (3.0)
Denver, Aurora, Lakewood (Colorado)	2015–2016	173 (3.0)	—	469 (9.6)	24 (3.3)
	2018–2019	198 (3.4)	37 (5.0)	537 (10.2)	28 (3.8)
Detroit, Warren, Dearborn (Michigan)	2015–2016	652 (8.1)	50 (4.5)	554 (7.0)	28 (2.5)
	2018–2019	639 (7.9)	44 (4.1)	580 (7.3)	30 (2.8)
Hartford, East Hartford, Middletown (Connecticut)	2015–2016	55 (2.5)	—	59 (2.5)	—
	2018–2019	46 (2.0)	—	91 (3.9)	—
Houston, The Woodlands, Sugar Land (Texas)	2015–2016	828 (6.1)	109 (5.6)	921 (8.2)	45 (2.3)
	2018–2019	817 (5.8)	143 (7.0)	936 (7.8)	50 (2.5)
Indianapolis, Carmel, Anderson (Indiana)	2015–2016	298 (7.7)	45 (8.3)	308 (8.9)	—
	2018–2019	325 (8.3)	57 (10.2)	361 (10.0)	23 (4.1)
Jacksonville (Florida)	2015–2016	208 (7.4)	32 (8.8)	299 (11.1)	—
	2018–2019	241 (8.4)	44 (11.7)	323 (11.4)	—
Kansas City (Missouri, Kansas)	2015–2016	327 (8.2)	38 (6.8)	375 (10.4)	22 (4.0)
	2018–2019	410(10.1)	51 (9.0)	471 (12.4)	31 (5.5)
Las Vegas, Henderson, Paradise (Nevada)	2015–2016	234 (5.6)	26 (4.8)	391 (10.4)	—
	2018–2019	224 (5.2)	36 (6.3)	468 (11.5)	—
Los Angeles, Long Beach, Anaheim (California)	2015–2016	1,003 (3.7)	123 (3.6)	781 (3.2)	25 (0.7)
	2018–2019	871 (3.3)	105 (3.2)	773 (3.2)	24 (0.7)
Louisville/Jefferson County (Kentucky, Indiana)	2015–2016	200 (8.4)	25 (7.9)	255 (11.1)	—
	2018–2019	189 (8.0)	27 (8.6)	252 (11.0)	—
Memphis (Tennessee, Mississippi, Arkansas)	2015–2016	396 (15.0)	52 (14.0)	183 (8.0)	—
	2018–2019	494 (18.9)	75 (20.5)	222 (9.5)	—
Miami, Fort Lauderdale, Pompano Beach (Florida)	2015–2016	669 (5.9)	98 (7.1)	613 (5.3)	—
	2018–2019	694 (6.0)	64 (4.6)	712 (5.9)	—
Milwaukee, Waukesha (Wisconsin)	2015–2016	267 (8.9)	30 (7.2)	182 (6.5)	—
	2018–2019	207 (7.1)	24 (5.8)	182 (6.4)	—
Minneapolis, St. Paul, Bloomington (Minnesota, Wisconsin)	2015–2016	136 (2.0)	26 (2.8)	315 (5.2)	20 (2.2)
	2018–2019	123 (1.8)	23 (2.4)	368 (5.7)	23 (2.4)
Nashville–Davidson, Murfreesboro, Franklin (Tennessee)	2015–2016	177 (4.8)	29 (6.1)	331 (10.3)	23 (4.9)
	2018–2019	230 (6.1)	45 (9.1)	348 (10.4)	20 (4.0)
New Orleans, Metairie (Louisiana)	2015–2016	404 (16.7)	54 (17.7)	186 (8.1)	—
	2018–2019	370 (15.6)	45 (14.6)	202 (8.7)	—
New York, Newark, Jersey City (New York, New Jersey, Pennsylvania)	2015–2016	917 (2.4)	91 (2.0)	513 (1.4)	—
	2018–2019	679 (1.8)	76 (1.7)	517 (1.4)	—
Oklahoma City (Oklahoma)	2015–2016	163 (6.0)	21 (5.7)	317 (13.5)	20 (5.5)
	2018–2019	151 (5.5)	—	319 (12.9)	—
Orlando, Kissimmee, Sanford (Florida)	2015–2016	251 (5.1)	23 (3.7)	275 (6.2)	—
	2018–2019	239 (4.5)	26 (4.0)	331 (7.0)	—
Philadelphia, Camden, Wilmington (Pennsylvania, New Jersey, Delaware, Maryland)	2015–2016	800 (6.8)	94 (6.1)	513 (4.5)	—
	2018–2019	849 (7.3)	96 (6.3)	526 (4.6)	—
Phoenix, Mesa, Chandler (Arizona)	2015–2016	397 (4.4)	42 (3.3)	865 (10.6)	34 (2.7)
	2018–2019	407 (4.3)	53 (4.0)	906 (10.2)	35 (2.6)
Pittsburgh (Pennsylvania)	2015–2016	233 (5.4)	38 (7.2)	381 (8.7)	—
	2018–2019	197 (4.5)	24 (4.7)	408 (9.1)	—
Portland, Vancouver, Hillsboro (Oregon, Washington)	2015–2016	80 (1.7)	—	356 (8.2)	—
	2018–2019	78 (1.6)	—	399 (8.7)	21 (3.6)
Providence, Warwick (Rhode Island, Massachusetts)	2015–2016	38 (1.1)	—	103 (3.3)	—
	2018–2019	32 (1.1)	—	96 (3.2)	—
Raleigh, Cary (North Carolina)	2015–2016	64 (2.5)	—	121 (5.4)	—
	2018–2019	55 (2.0)	—	152 (6.3)	—
Richmond (Virginia)	2015–2016	178 (7.3)	—	211 (9.0)	—
	2018–2019	189 (7.7)	25 (7.9)	205 (8.8)	—
Riverside, San Bernardino, Ontario (California)	2015–2016	303 (3.3)	41 (3.0)	408 (5.4)	20 (1.5)
	2018–2019	366 (4.0)	43 (3.2)	455 (5.7)	—
Sacramento, Roseville, Folsom (California)	2015–2016	162 (3.6)	21 (3.5)	259 (6.2)	—
	2018–2019	135 (2.9)	—	237 (5.4)	—
St. Louis (Missouri, Illinois)	2015–2016	596 (11.4)	61 (8.6)	442 (8.7)	—
	2018–2019	676 (13.0)	82 (11.8)	486 (9.4)	23 (3.3)
Salt Lake City (Utah)	2015–2016	46 (1.9)	—	237 (12.4)	20 (5.7)
	2018–2019	44 (1.8)	—	246 (12.0)	—
San Antonio, New Braunfels (Texas)	2015–2016	266 (5.5)	27 (3.9)	305 (7.3)	20 (2.9)
	2018–2019	246 (4.9)	42 (5.8)	380 (8.7)	36 (5.0)
San Diego, Chula Vista, Carlsbad (California)	2015–2016	103 (1.6)	—	282 (4.8)	—
	2018–2019	107 (1.5)	—	335 (5.5)	—
San Francisco, Oakland, Berkeley (California)	2015–2016	414 (4.5)	60 (5.8)	263 (3.0)	—
	2018–2019	285 (3.1)	33 (3.2)	264 (3.0)	—
San Jose, Sunnyvale, Santa Clara (California)	2015–2016	58 (1.5)	—	97 (2.7)	—
	2018–2019	43 (1.1)	—	99 (2.7)	—
Seattle, Tacoma, Bellevue (Washington)	2015–2016	165 (2.2)	32 (3.6)	452 (6.7)	29 (3.3)
	2018–2019	186 (2.4)	37 (4.1)	523 (7.4)	32 (3.5)
Tampa, St. Petersburg, Clearwater (Florida)	2015–2016	204 (3.7)	21 (3.1)	568 (9.4)	—
	2018–2019	213 (3.7)	23 (3.2)	591 (9.4)	—
Virginia Beach, Norfolk, Newport News (Virginia, North Carolina)	2015–2016	248 (6.8)	38 (8.7)	271 (8.3)	20 (4.6)
	2018–2019	247 (7.0)	35 (8.0)	295 (9.3)	—
Washington, Arlington, Alexandria (DC, Virginia, Maryland, West Virginia)	2015–2016	471 (3.8)	42 (2.7)	459 (4.3)	28 (1.8)
	2018–2019	509 (4.1)	72 (4.6)	471 (4.2)	29 (1.8)

**Abbreviations:** DC = District of Columbia; MSA = metropolitan statistical area.

* Numbers and rates reflect decedent place of residence, not place of occurrence. This table includes only the 50 most populous MSAs among the 384 MSAs currently delineated and therefore cannot be used to establish comprehensive national rankings.

^†^ These national and MSA–specific numbers exclude a small fraction of records with undocumented decedent age (four firearm homicides and four firearm suicides) and might therefore differ slightly from numbers in the text.

^§^ Per 100,000 residents per year. Rates are age–adjusted to the year 2000 U.S. standard population profile.

^¶^ Dashes indicate entry suppressed because of statistical instability or data confidentiality concerns (both associated with small numbers).

Overall firearm suicide rates during 2018–2019 also varied widely across large MSAs, ranging from 1.4 (the New York, Newark, Jersey City [New York, New Jersey, Pennsylvania] MSA) to 12.9 (the Oklahoma City [Oklahoma] MSA) per 100,000 residents per year (Table). The rates for large MSAs combined and nationally were 6.2 and 8.1, respectively, both representing increases from 2015–2016, when the respective rates were 5.8 and 7.7. From 2015–2016 to 2018–2019, firearm suicides rates increased for 30 (60%) of the 50 largest MSAs. During both periods, youth firearm suicide rates were much lower than overall rates. For the largest MSAs collectively, the youth firearm suicide rate during 2018–2019 was 2.0, comparable to the rate of 1.9 observed for 2015–2016; the national rate for 2018–2019 was 2.9, representing a more notable increase from the rate of 2.5 for the earlier period. Similar to firearm homicides, males accounted for approximately 85% of firearm suicides in both reporting periods for the 50 largest MSAs combined and nationally (National Vital Statistics System, unpublished data, 2020). During both periods, non-Hispanic White (White) persons represented a disproportionately large percentage of firearm suicide victims (approximately 80%) for the largest MSAs combined relative to population representation (approximately 55%), with a comparable national pattern (85% and 63%, respectively) (National Vital Statistics System, unpublished data, 2020).

## Discussion

During 2018–2019, homicide was the sixteenth leading cause of death overall in the United States and the third leading cause among youths (*2*); firearm injuries were the underlying cause of death in 75% of all homicides and in 91% of youth homicides (*1*). From 2015–2016 to 2018–2019, firearm homicide rates among persons of all ages were nearly unchanged nationally and for the 50 largest MSAs combined. The youth firearm homicide rate for the largest MSAs combined increased somewhat across the two periods, with the national rate among youths increasing more notably; these increases coincided with those in youth firearm homicide rates for less populous metropolitan and nonmetropolitan areas which markedly exceeded the increase for the largest MSAs (*3*). For the largest MSAs collectively, firearm homicide rates among persons of all ages and among youths have both remained higher than corresponding national rates.

For the same period, suicide was the tenth leading cause of death nationwide among persons aged ≥10 years and the second leading cause among youths (*2*); firearm injuries were listed as the underlying cause of death in 50% of all suicides and in 43% of all youth suicides (*1*). Previously observed increases in overall firearm suicide rates continued in recent years, in the largest MSAs collectively and nationally. Youth firearm suicide rates also increased nationally but remained nearly level in the largest MSAs combined; this coincided with increases in youth rates for less populous metropolitan and nonmetropolitan areas, with the rate for nonmetropolitan areas increasing most notably (*3*). In contrast to firearm homicide rates, overall firearm suicide rates and youth firearm suicide rates in the largest MSAs collectively have remained lower than the corresponding national rates.

Firearm homicide remains a persistent problem in metropolitan areas in the Unites States, especially among young Black males, and increasingly, in less populous and nonmetropolitan areas as well. Previous research has found that wealth inequality, lack of trust in institutions, and economic deprivation are associated with firearm homicide rates at the county level (*4*). Persistently high rates among racial and ethnic minority youths might be rooted in stressors associated with living in under-resourced communities and ultimately caused by systemic racism or multigenerational poverty resulting from limited educational and economic opportunities (*5*). Although not specifically evaluated for effects on firearm homicide, prevention efforts that strengthen household financial security (e.g., tax credits, child care subsidies, temporary assistance to families, and livable wages) and that improve access to high-quality early childhood education have demonstrated positive effects on important risk factors for firearm homicide, including poverty, school performance, school dropout rates, substance use, behavioral problems, and arrests for violent and nonviolent offenses (*6*,*7*).

Firearm suicide similarly remains a persistent public health problem, particularly among White males. Multiple factors influence suicide risk, including family and relationship problems, job and financial concerns, mental illness, substance use, and stigma around help-seeking (*8*,*9*). The effects of the evolving drug overdose epidemic might also be contributing to the risk among youths, either directly through their own substance use or indirectly through adult use that increases the prevalence of adverse childhood experiences (*6*).

Another factor likely affecting both firearm homicide and suicide is access to firearms by persons at risk for harming themselves or others (*10*). However, the specific nature of and avenues to firearm access among inner-city youths should be more fully explored. Reducing access to lethal means before or during an acute suicidal crisis by safely storing firearms or temporarily removing them from the home can help reduce suicide risk, particularly among youths (*9*).

A focus on upstream prevention can potentially reduce both firearm homicide and suicide rates. This includes approaches that prevent the risk of firearm homicide and suicide in the first place, such as strengthening economic supports, strengthening access to and delivery of care, teaching coping and problem-solving skills, building positive and nurturing relationships, connecting youths to caring adults and activities, and implementing place-based interventions (e.g., remediating abandoned buildings and blighted areas, creating and maintaining green spaces, and investing in basic services and commercial activities) (*6*,*7*,*9*). Together, such measures are associated with reductions in youth violence and crime, suicide, and risk factors such as weapon carrying, substance use, school dropout, involvement in high-risk social networks such as gangs, depression, stress and anxiety, and suicidal thoughts and behavior (*7*,*9*). As part of a comprehensive prevention approach, individual persons and communities at increased risk should be supported through identification of and response to warning signs, through evidence-based programs and treatment (*6*,*9*), and by lessening harms after violence and suicide have occurred (*6*,*7*,*9*).

The findings in this report are subject to at least two limitations. First, although the findings incorporate the most recent comprehensive mortality data available at the time of analysis, they do not fully characterize changing patterns in firearm-related violence; summary statistics for 2020 indicate a further increase in the rate of firearm-related death overall, largely because of an increase in the homicide rate (*3*). Second, and notwithstanding the intended focus on youth firearm homicide and suicide, a broader analysis might have addressed these outcomes for other age groups not separately considered.

Firearm injuries contribute substantially to premature death and disability. Ongoing monitoring of such injuries in both metropolitan and nonmetropolitan areas can help assess and guide prevention efforts.

**Summary**

**What is already known about this topic?**

In the United States, firearm homicides are disproportionately concentrated in large metropolitan areas, and firearm suicides are disproportionately concentrated outside such areas.

**What is added by this report?**

Firearm homicide rates among persons of all ages remained nearly unchanged from 2015–2016 to 2018–2019 in large metropolitan areas collectively and nationally; rates among youths increased somewhat within and outside large metropolitan areas. Firearm suicide rates increased in large metropolitan areas collectively and nationally; the rate among youths increased outside large metropolitan areas.

**What are the implications for public health practice?**

There is an urgent need for comprehensive firearm homicide and suicide prevention efforts to reduce overall rates as well as ethnic and racial disparities; increases in rates among youths within and outside of metropolitan areas represent a notable concern.

## Footnotes

* https://www.cdc.gov/mmwr/preview/mmwrhtml/mm6018a1.htm; https://www.cdc.gov/mmwr/preview/mmwrhtml/mm6230a1.htm; https://www.cdc.gov/mmwr/volumes/67/wr/mm6744a3.htm

† An MSA is defined by the U.S. Office of Management and Budget (OMB) as consisting of “at least one urbanized area of 50,000 or more population, plus adjacent territory that has a high degree of social and economic integration with the core as measured by commuting ties.” MSAs are geographically delineated by groupings of neighboring counties and can cross state boundaries; names are assigned by the OMB based on the names of one to three principal cities or places within each MSA. This report refers to MSAs as delineated by the OMB in March 2020.

§ The same MSAs were the 50 most populous during both reporting periods; rankings by total population changed slightly. This group includes most MSAs with a resident population of at least 1 million and represented approximately 55% of the U.S. resident population during 2018–2019.
